# Depression and Suicidality in Patients with Left Ventricular Assist Devices and Advanced Cardiac Therapies: Mechanisms, Risk Factors, and Clinical Management

**DOI:** 10.3390/medsci14020244

**Published:** 2026-05-07

**Authors:** Vasileios Leivaditis, Francesk Mulita, Chrysa Andrikopoulou, Ejona Shaska, Elias Liolis, Sofoklis Mitsos, Konstantinos Grapatsas, Periklis Tomos, Nikolaos G. Baikoussis

**Affiliations:** 1 Department of Cardiothoracic and Vascular Surgery, Westpfalz Klinikum, 67655 Kaiserslautern, Germany; vleivaditis@westpfalz-klinikum.de; 2 Department of General Surgery, General Hospital of Eastern Achaia–Unit of Aigio, 25100 Aigio, Greece; chrysa661@gmail.com; 3 Department of Psychiatry, “Ali Mihali” Psychiatric Hospital, 9401 Vlora, Albania; zilja.jona@yahoo.it; 4 Department of Oncology, General University Hospital of Patras, 26504 Patras, Greece; iliolis@upnet.gr; 5 Department of Thoracic Surgery, Attikon General Hospital, National and Kapodistrian University of Athens, 12462 Athens, Greece; sophocmit@yahoo.gr (S.M.); periklistomos@hotmail.com (P.T.); 6 Department of Thoracic Surgery and Thoracic Endoscopy, Ruhrlandklinik, West German Lung Center, University Hospital Essen, University Duisburg-Essen, 45141 Essen, Germany; grapatsaskostas@gmail.com; 7 Department of Cardiac Surgery, Ippokrateio General Hospital of Athens, 11527 Athens, Greece; nikolaos.baikoussis@gmail.com

**Keywords:** left ventricular assist device (LVAD), advanced heart failure, depression, suicidality, mechanical circulatory support, psychosocial factors, mental health, cardiac surgery

## Abstract

Background: The increasing use of advanced cardiac surgical therapies, particularly left ventricular assist devices (LVADs), has improved survival in patients with end-stage heart failure. However, the psychological burden associated with these therapies—especially depression and suicidality—remains underrecognized. Objectives: This narrative review synthesizes current evidence on the prevalence, underlying mechanisms, risk factors, screening strategies, and management of depression and suicidality in patients undergoing LVAD implantation and other advanced cardiac surgical interventions. Methods: A structured literature search of PubMed, Embase, and Scopus was conducted for studies published between 2020 and 2025 addressing depression, suicidal ideation, suicide attempts, and psychological distress in LVAD and advanced cardiac therapy populations. Results: Depression affects approximately 20–42% of patients with advanced heart failure, including those supported with LVADs, while suicidal ideation is reported in up to 12% of LVAD recipients, with higher rates of suicide attempts compared to other chronic disease populations. Risk factors are multifactorial and can be categorized into patient-related, disease-related, device-related, and psychosocial domains. Proposed mechanisms include neurohormonal dysregulation, systemic inflammation, and psychological processes such as loss of autonomy and existential distress. Although validated screening tools and multidisciplinary management strategies are available, their implementation in routine clinical practice remains inconsistent. Conclusions: Depression and suicidality represent significant and complex challenges in patients undergoing advanced cardiac therapies, particularly LVAD support. Systematic mental health screening and integrated, multidisciplinary care models are essential to improve patient outcomes. Future research should focus on longitudinal assessment, standardized suicide risk monitoring, and the development of targeted, evidence-based interventions for this vulnerable population.

## 1. Introduction

### 1.1. Burden of Advanced Heart Failure

Heart failure (HF) represents a major global health challenge with substantial clinical and economic impact. According to the 2024 statistics report by the Heart Failure Society of America, approximately 6.7 million adults in the United States are currently living with HF, a number projected to rise to 8.7 million by 2030 and to exceed 11 million by 2050. Overall, it is estimated that one in four individuals will develop HF during their lifetime [1].

Globally, the burden of HF is similarly substantial. In 2021, an estimated 55.5 million people were living with HF worldwide, more than doubling from 25.4 million in 1990, underscoring the rapidly increasing prevalence of the disease [2]. Community-based studies suggest that from approximately 5.1% to 7.7% of all HF patients meet the criteria for advanced HF at any given time. Advanced HF is characterized by persistent symptoms at rest or with minimal exertion despite optimal medical and device therapy, recurrent hospitalizations, and markedly impaired quality of life [3].

The management of advanced HF has evolved significantly in recent years, with the development of advanced cardiac surgical therapies for patients who do not respond adequately to guideline-directed medical treatment. Left ventricular assist devices (LVADs) are surgically implanted mechanical pumps designed to support cardiac output by augmenting left ventricular function. LVADs may be used as a bridge to transplantation (BTT), as destination therapy (DT) for patients ineligible for transplantation, or as a bridge to decision or recovery [4].

According to the Society of Thoracic Surgeons (STS) INTERMACS 2025 Annual Report, more than 28,000 continuous-flow LVADs were implanted in the United States between 2015 and 2024 [5]. Technological advancements have led to the near-universal adoption of fully magnetically levitated devices, which accounted for approximately 99.8% of implants in 2022, with the HeartMate 3 emerging as the predominant device in current clinical practice.

In addition to LVAD therapy, patients with advanced HF may be candidates for heart transplantation, temporary mechanical circulatory support modalities such as intra-aortic balloon pump (IABP) and extracorporeal membrane oxygenation (ECMO), as well as complex surgical interventions including ventricular reconstruction [6].

### 1.2. Psychological Burden of Severe Cardiovascular Disease

There is a well-established bidirectional relationship between cardiovascular disease and mental health. Patients with heart failure (HF) are approximately four to five times more likely to experience depression compared with age-matched individuals in the general population [7]. More recent pooled estimates indicate that up to 24.7% of HF patients are affected by depression globally.

The prevalence of depression increases with the severity of HF. Reported rates range from approximately 11% in patients with New York Heart Association (NYHA) Class I disease to as high as 42% in those with NYHA Class IV disease [8]. This gradient highlights the close relationship between disease burden and psychological distress.

Importantly, depression in HF is not merely a coexisting psychological condition but an independent predictor of adverse clinical outcomes. It has been associated with accelerated disease progression, increased hospital readmissions, reduced adherence to medical therapy, and higher all-cause mortality, particularly in patients with reduced ejection fraction [9].

More broadly, chronic medical illness is a significant risk factor for suicidal ideation and behavior. Among hospitalized patients, hopelessness related to physical illness has been identified as one of the strongest predictors of suicidal ideation [10].

### 1.3. Unique Challenges of Advanced Cardiac Surgical Therapies

Patients undergoing LVAD implantation or other advanced cardiac surgical therapies face a distinct set of psychological stressors that differ from those encountered in other chronic medical conditions. Living with a life-sustaining mechanical device fundamentally alters daily existence. Patients must continuously manage an external apparatus while coping with the constant risk of mechanical failure and device-related complications, including infections, driveline complications, and thromboembolic events [11].

Lifestyle restrictions imposed by device therapy—such as limitations on bathing, physical activity, and travel—can significantly impact patients’ autonomy and sense of identity [12]. In addition, the surgical experience itself, often involving prolonged intensive care unit (ICU) stays, may precipitate or exacerbate underlying psychiatric vulnerability. Patients frequently develop substantial dependence on caregivers, and most LVAD programs require the continuous availability of a trained caregiver. This requirement places a considerable emotional and practical burden on both patients and their families [13].

Furthermore, frequent and unpredictable device alarms can contribute to persistent anxiety and fear, reinforcing a constant awareness of the life-threatening nature of the condition. Collectively, these factors create a substantial psychosocial burden that often exceeds that observed in typical chronic illnesses.

### 1.4. Rationale for This Review

Cardiovascular disease remains the leading cause of death worldwide, according to the World Health Organization. In parallel with the increasing use of advanced cardiac therapies, the associated psychological burden—particularly depression and suicidality—has become increasingly relevant but remains underrecognized and insufficiently studied.

Current evidence on the prevalence, determinants, and clinical implications of suicidal behavior in LVAD recipients is limited. Moreover, there is a lack of comprehensive reviews that integrate existing knowledge on depression and suicidality across the spectrum of advanced cardiac surgical therapies. This gap is particularly important given the unique ethical challenges associated with device-dependent patients, including the potential for self-harm through device disconnection.

Accordingly, a comprehensive synthesis of the available literature is urgently needed to better understand the scope of the problem and to inform clinical practice and future research.

### 1.5. Aim of This Review

This review aims to synthesize current evidence on the epidemiology, pathophysiological and psychosocial mechanisms, risk factors, clinical implications, screening strategies, and management of depression and suicidality in patients undergoing LVAD implantation and other advanced cardiac surgical therapies. By integrating these domains, the review seeks to inform clinical practice and identify key priorities for future research.

## 2. Methods

### 2.1. Literature Search Strategy

A structured literature search was conducted in the PubMed, Embase, and Scopus databases, covering publications from January 2020 to December 2025. Search terms were used in various combinations and included: “left ventricular assist device,” “LVAD,” “mechanical circulatory support,” “ventricular assist device,” “heart transplantation,” “cardiac surgery,” “advanced heart failure,” “depression,” “depressive disorder,” “suicidal ideation,” “suicide,” “suicide attempt,” “suicidality,” “mental health,” “psychological distress,” “anxiety,” “psychosocial,” “quality of life,” and “psychiatric outcomes.”

The reference lists of retrieved articles were also screened to identify additional relevant studies. In addition, the 2025 European Society of Cardiology (ESC) Clinical Consensus Statement on Mental Health and Cardiovascular Disease was consulted as an authoritative clinical guideline [14].

### 2.2. Inclusion and Exclusion Criteria

Studies were eligible for inclusion if they involved adult patients (≥18 years) with advanced heart failure (HF) who had undergone, or were being evaluated for, LVAD implantation, heart transplantation, temporary mechanical circulatory support, or complex cardiac surgical procedures, and if they assessed depression, suicidal ideation, suicide attempts, or other relevant psychological outcomes. Studies were included regardless of design.

Eligible study types included observational studies, cohort studies, case reports, clinical trials, systematic reviews, and narrative reviews. Articles were excluded if they focused exclusively on pediatric or congenital heart disease populations, did not report original data or a relevant synthesis aligned with the aims of this review, or were not published in English.

### 2.3. Study Selection and Quality Considerations

Study selection was conducted through a structured process involving screening of titles, abstracts, and full-text articles based on predefined inclusion and exclusion criteria. To minimize selection bias, the screening process was performed independently by two authors, with discrepancies resolved through discussion and consensus.

Although this study is a narrative review and did not include a formal quantitative synthesis, attention was given to the methodological quality of the included studies. In particular, greater emphasis was placed on higher-quality evidence, including large cohort studies, registry analyses, and systematic reviews. Formal quality assessment tools, such as the Newcastle–Ottawa Scale (NOS) or Joanna Briggs Institute (JBI) criteria, were not systematically applied; however, study quality and risk of bias were considered during data interpretation and synthesis.

**Figure 1:**
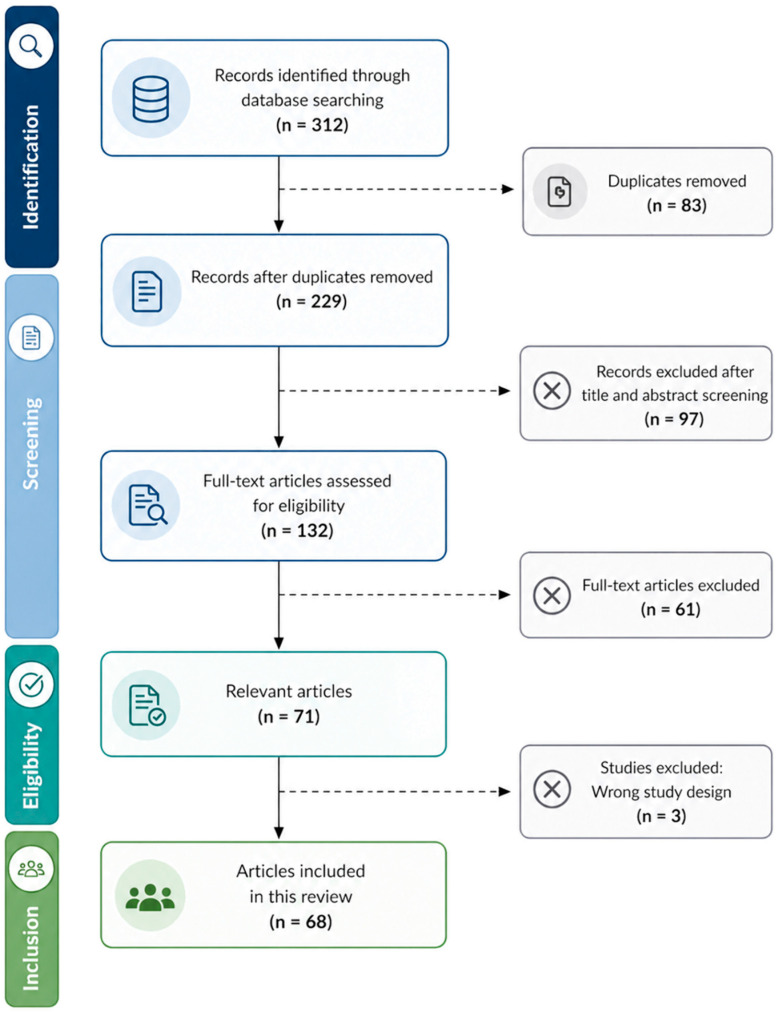
Literature selection process. Flowchart illustrating the identification, screening, eligibility assessment, and inclusion of studies in this narrative review. Records were identified through database searching (PubMed, Embase, and Scopus) followed by duplicate removal, title and abstract screening, and full-text assessment. Studies not meeting the inclusion criteria were excluded at each stage. A total of 68 articles were included in the final qualitative synthesis.

This narrative review was conducted in accordance with the principles of the Preferred Reporting Items for Systematic Reviews and Meta-Analyses (PRISMA) guidelines, where applicable, to enhance transparency and methodological rigor. The study selection process is illustrated in Figure 1. Following database searching and removal of duplicates, records were screened based on title and abstract, and subsequently assessed for eligibility through full-text review. Studies not meeting the predefined inclusion criteria were excluded at each stage. A total of 68 articles were ultimately included in the qualitative synthesis.

In addition, during evidence interpretation, study quality was considered in a pragmatic and narrative manner. Greater weight was given to studies with larger sample sizes, prospective or multicenter designs, and those utilizing validated assessment instruments for depression and suicidality. Registry-based analyses and systematic reviews were also prioritized when available. Conversely, findings from smaller, single-center studies or case reports were interpreted with appropriate caution, particularly where methodological limitations or potential sources of bias were identified. This approach aimed to enhance the robustness and clinical relevance of the overall synthesis while acknowledging the inherent limitations of the available evidence.

### 2.4. Narrative Synthesis

Given the substantial heterogeneity among the included studies—particularly with respect to patient populations, duration of follow-up, assessment instruments for depression, and outcome definitions—a formal meta-analytic synthesis was neither feasible nor appropriate. Therefore, findings were synthesized qualitatively, with emphasis on the most robust and recent evidence, to provide a coherent and clinically relevant overview. Where available, primary studies were used to extract effect sizes and prevalence estimates to support the narrative synthesis.

All graphical illustrations in this manuscript are original and were developed by the authors using standard, vector-based, digital illustration software (Adobe Illustrator, Acrobat Reader X 10.1.4, Adobe Inc., San Jose, CA, USA) to summarize and conceptualize the reviewed literature and are intended to provide conceptual summaries of the discussed mechanisms. They are intended as conceptual schematic models to illustrate and summarize the mechanisms and clinical frameworks discussed in the manuscript and do not represent primary data.

## 3. Psychological Burden in Advanced Heart Failure

### 3.1. Prevalence of Depression in Heart Failure

The association between depression and heart failure (HF) has long been recognized; however, its clinical significance remains underappreciated. A 2025 meta-analysis of 39 studies involving 63,444 patients with cardiovascular disease reported an overall depression prevalence of 20.8%, with a higher prevalence of 24.7% observed specifically among patients with HF [7].

The prevalence of depression increases with disease severity. According to Polikandrioti and Tsami [8], patients with advanced HF—those most likely to be candidates for LVAD implantation—exhibit markedly higher rates of depression, ranging from approximately 11% in New York Heart Association (NYHA) Class I to 42% in Class IV. These findings underline that depression in HF is not merely a reactive phenomenon but a comorbidity with significant prognostic implications.

Data from Danish national registries further demonstrate that patients with HF and a history of depression have higher all-cause mortality compared with those without depression, with rates of 36% versus 33% at 1 year and 68% versus 63% at 5 years. This increased risk is particularly pronounced in patients with reduced ejection fraction [15].

In addition, depression in HF is associated with poorer medication adherence, reduced engagement in self-care behaviors, and increased rates of emergency hospitalizations, all of which contribute to accelerated disease progression and diminished quality of life [9].

### 3.2. Depression and Suicidality in Chronic Illness

Living with a chronic physical illness is associated with an increased risk of suicidal ideation and behavior. A recent large-scale epidemiological study demonstrated that most major chronic disease categories are linked to poorer mental health and a higher risk of suicide. Among the identified factors, subjective perception of poor health showed the strongest association with suicidal thoughts and attempts [16].

Hopelessness, a core cognitive feature of depression, represents a key psychological link between chronic illness burden and suicidality. A network analysis of comorbid psychological distress and suicidal behavior identified hopelessness and suicidal ideation as central bridging symptoms between these domains. Furthermore, sleep disturbance, anxiety, and poor social relationships were found to influence suicidal ideation primarily through the mediating effect of hopelessness [17].

Among hospitalized patients, a hopeless outlook regarding one’s medical condition is associated with more than a fivefold increase in the likelihood of a positive suicide risk screen (adjusted OR: 5.69; 95% CI: 2.52–12.64). Additionally, the presence of chronic pain approximately doubles this risk [10]. These findings have important clinical implications for patients with advanced HF, who frequently experience both persistent symptoms and psychological distress. Reported prevalence rates of depression and suicidality across advanced cardiac therapy populations are summarized in Table 1 [18,19,20,21,22,23,24,25,26,27,28,29,30,31,32,33,34,35,36,37,38,39,40,41].

Depression prevalence varies depending on population characteristics, timing of assessment, and diagnostic instruments used. Suicidality remains underreported across studies, with limited standardized assessment and potential misclassification, particularly in device-dependent populations such as LVAD recipients.

### 3.3. Psychiatric Comorbidity Before Advanced Therapy

A substantial proportion of patients referred for LVAD implantation or cardiac transplantation present with pre-existing psychiatric diagnoses or significant psychological distress at the time of evaluation.

Analysis of the INTERMACS registry by DeFilippis et al. [18] demonstrated that, among 15,403 patients who received a continuous-flow LVAD, 20.5% had at least one psychosocial risk factor prior to implantation. The most common was substance abuse (12.6%), followed by limited social support and psychiatric comorbidity, including major depressive disorder.

Importantly, a history of mental illness at the time of HVAD implantation was associated with significantly increased post-implant risks, including device-related infection, gastrointestinal bleeding, pump thrombosis, and hospital readmission [18]. These findings demonstrate the importance of comprehensive preoperative psychiatric assessment, not merely as a gatekeeping tool, but as a means of identifying modifiable risk factors and informing individualized psychosocial support and risk management strategies.

## 4. Lvad Therapy and Psychological Outcomes

### 4.1. Overview of LVAD Therapy

A left ventricular assist device (LVAD) is a surgically implanted mechanical pump designed to support cardiac output in patients with advanced heart failure. The device withdraws blood from the left ventricle and continuously pumps it into the aorta, thereby augmenting systemic circulation. Contemporary third-generation devices, such as the fully magnetically levitated HeartMate 3, have significantly improved survival rates and reduced adverse events compared with earlier axial-flow devices [19].

In 2022, approximately 2517 primary LVAD implantations were performed in the United States, of which 99.8% utilized magnetically levitated technology [20]. LVAD therapy serves multiple clinical purposes. It may be used as a bridge to transplantation (BTT) in patients awaiting heart transplantation, as destination therapy (DT) in patients who are not transplant candidates, or as a bridge to decision when prognosis or transplant eligibility remains uncertain. In selected cases, LVADs may also be employed as a bridge to recovery in potentially reversible cardiomyopathies [20].

Although pediatric heart transplantation is increasingly driven by congenital heart disease in many centers [21], the use of LVADs remains primarily focused on adult populations with advanced HF. Following implantation, patients require meticulous postoperative management, including lifelong anticoagulation, continuous monitoring of device function, driveline care, and close follow-up by a specialized multidisciplinary team [22].

### 4.2. Prevalence of Depression in LVAD Patients

A high prevalence of depression has been consistently reported among LVAD recipients, both in the perioperative period and during long-term device support. However, the trajectory of depressive symptoms is dynamic and heterogeneous over time. In a study of 120 LVAD recipients with a median follow-up of approximately 0.82 years post-implantation, 15% of patients were found to have moderate-to-severe depression based on self-reported measures, while 12% reported suicidal ideation [23].

Depressive symptoms are often more pronounced prior to implantation. Alnsasra et al. [23] highlighted the elevated burden of pre-implant depression, and Yost et al. [24] reported that 41% of LVAD candidates had Beck Depression Inventory-II (BDI-II) scores consistent with moderate depression before implantation. This proportion decreased to 18% at six to twelve months following LVAD placement, suggesting an initial improvement in psychological status.

Longitudinal data further indicate that sleep disturbance—both a symptom and a contributing factor to depression—is common before and after LVAD implantation and is significantly associated with higher depression scores [25]. Over time, the clinical course becomes more complex. Many patients experience improvements in mood and quality of life as their physical condition stabilizes [26]. However, long-term data remain limited, and depressive symptoms may recur or worsen beyond the first year, particularly in patients receiving destination therapy (DT), where lifelong device dependence and cumulative medical complications contribute to psychological burden [23].

This pattern is often described as a “honeymoon phase,” during which initial relief from debilitating symptoms such as dyspnea is followed by challenges in long-term psychological adaptation [26]. Monitoring the temporal evolution of anxiety and depression is therefore essential, as it may help identify patients at increased risk of adverse clinical outcomes and major cardiovascular events [27].

**Figure 2:**
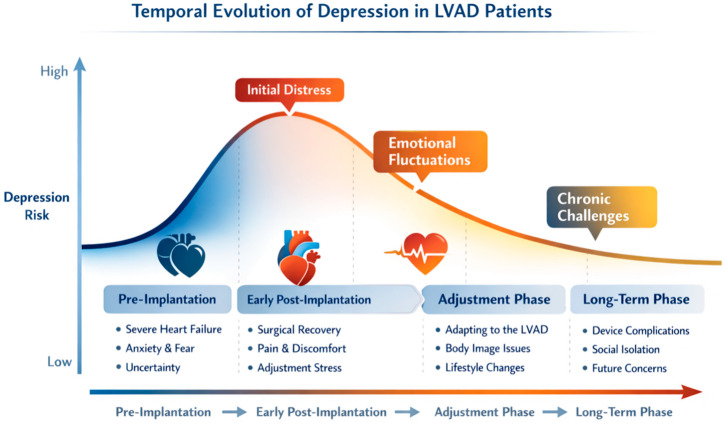
Temporal evolution of depression and psychological distress in patients undergoing LVAD implantation. Depressive symptoms in patients with advanced heart failure are typically elevated prior to LVAD implantation, reflecting severe disease burden, uncertainty, and psychological distress. Following implantation, many patients experience an initial improvement in mood and quality of life (“honeymoon phase”) associated with relief of physical symptoms. However, during the intermediate phase, patients often encounter emotional fluctuations related to adaptation to device dependence, body image changes, and lifestyle restrictions. In the long term, depressive symptoms may persist or recur, particularly in destination therapy patients, driven by chronic device burden, complications, social isolation, and ongoing existential concerns. This dynamic trajectory highlights the need for continuous psychological monitoring across all stages of care. This figure represents a conceptual schematic model and does not depict primary data.

Although LVAD therapy has been shown to improve physical function and reduce hospitalizations, mental health challenges—including the burden of ongoing self-care, lifestyle restrictions, and complex treatment regimens—remain persistent and clinically significant, necessitating continuous psychological support [28]. The temporal trajectory of depressive symptoms in LVAD patients across the continuum of care is illustrated in Figure 2.

### 4.3. Suicidal Ideation and Suicide in LVAD Patients

Patients with LVAD support, similar to the general population, may experience suicidal ideation and behavior; however, in this population, suicidality represents one of the most urgent and ethically complex clinical challenges. The ASSIST-ICD study, a French multicenter cohort including 659 LVAD recipients across 19 centers between 2006 and 2016, provides landmark data in this field [29].

Among 494 patients discharged from hospital, 10 (2%) attempted or completed suicide over a median follow-up of 18.8 months [29]. Notably, eight of these attempts were fatal and involved methods such as drug intoxication, driveline section, and battery disconnection—means that are uniquely accessible to device-dependent patients. This rate is markedly higher than the annual suicide attempt rate reported in the general population in France (approximately 0.03%) and in patients with other chronic diseases (approximately 0.06%).

Importantly, only two of the ten patients who attempted or completed suicide had a documented psychiatric history, suggesting that LVAD-related factors alone may significantly contribute to suicide risk. Nevertheless, the majority of these patients (8/10) had exhibited psychiatric symptoms such as sadness, social isolation, or hopelessness prior to the event. In addition, only 20% of patients in this group were followed in centers with an LVAD coordinator, compared with 60.5% in those without suicidal behavior, highlighting the potential protective role of structured follow-up [29].

Further evidence from case reports underscores the clinical complexity of suicidality in this population. Jiménez-Blanco Bravo et al. [30] described a destination therapy (DT) LVAD patient who attempted suicide in the context of psychosocial stressors related to the COVID-19 pandemic, despite having no prior psychiatric history. These findings emphasize the importance of ongoing psychological assessment and long-term support beyond the pre-implant evaluation phase.

Consistent with these observations, Alnsasra et al. [23] reported that suicidal ideation was present in approximately 12% of LVAD recipients. The availability of device-specific means of self-harm, such as device disconnection, introduces an additional dimension of suicide risk that is not encountered in other chronic disease populations.

Finally, a 2024 comparative analysis of LVAD and cardiac allograft recipients demonstrated distinct psychiatric comorbidity profiles between these groups, further supporting the need for therapy-specific mental health assessment and management strategies [31].

### 4.4. Psychological Adaptation to Device Dependence

The psychological experience of living with an LVAD is profoundly transformative, requiring patients to adapt to a fundamentally altered sense of bodily integrity and daily existence. This adjustment often involves an ongoing process of redefining identity and autonomy in the context of device dependence. A phenomenological study by Rapelli et al. [32], exploring the lived experiences of LVAD patient–caregiver dyads, identified four overarching themes: “being between life and death,” “being a human with a steel heart,” “sharing is caring (and burden),” and “being small and passive.” These findings highlight the ambivalent nature of the LVAD experience, characterized by simultaneous gratitude for survival and grief for the pre-illness self [32].

Body image disturbance represents a significant component of this psychological adaptation. In a 2025 cross-sectional study of 54 LVAD recipients, Milaniak et al. [33] found that anxiety, depressive symptoms, and post-implant complications were significant predictors of negative body image, with many patients struggling to integrate the external driveline into their sense of self.

Fear is another central aspect of the psychological burden. A systematic review of patients with advanced HF identified fear of disease progression as the predominant concern, closely associated with both depression and anxiety. In LVAD recipients, fears extend beyond disease progression to include device-related concerns, transplantation uncertainty, and broader psychological and existential stressors [34].

The complexity of adaptation is further illustrated in a qualitative study by Levelink et al. [35], which identified a wide range of psychosocial, health-related, and treatment-related burdens among patients receiving destination therapy (DT) LVAD support. The study also described multiple coping strategies employed by patients over time, ranging from three months to more than ten years post-implantation. This variability underlines the heterogeneity of patient experiences and highlights the need for individualized psychosocial support strategies.

## 5. Mental Health in Other Advanced Cardiac Surgical Therapies

### 5.1. Heart Transplantation

Heart transplantation remains the gold standard treatment for eligible patients with end-stage heart failure (HF), but it is associated with significant psychological challenges. A cross-sectional meta-analysis including 2169 heart transplant recipients reported substantial rates of anxiety (11.1%), depression (21.6%), and post-traumatic stress disorder (PTSD) (13.5%) [36].

The heart carries unique symbolic and emotional significance compared with other organs, often perceived as central to identity and emotional experience. As a result, psychological adaptation following transplantation involves complex processes related to the integration of a donor organ into one’s sense of self [37].

In a retrospective single-center cohort study, De la Rosa et al. [38] found that post-transplant depression was associated with a more complicated clinical course, including higher rates of rejection and infection. In contrast, pre-transplant depression did not independently predict outcomes, suggesting that ongoing monitoring for de novo depression after transplantation is at least as important as preoperative psychiatric assessment.

The concept of “psychologically informed heart transplantation care,” as proposed by Assabiny et al. [39], emphasizes the critical role of psychosocial factors in influencing adherence to complex immunosuppressive regimens, psychological stability, and social support. This framework advocates for a fully integrated, multidisciplinary care model throughout the transplant continuum.

Additionally, approximately 10% of heart transplant recipients experience PTSD following intensive care treatment, with symptoms including intrusive memories, flashbacks, nightmares, and avoidance behaviors [36]. These findings underscore the importance of systematic psychological assessment and support in this population.

### 5.2. Temporary Mechanical Circulatory Support and ECMO

Survivors of extracorporeal membrane oxygenation (ECMO), a form of temporary mechanical circulatory support used in critically ill patients, frequently experience a substantial psychological burden following hospital discharge. In a large Canadian population-based cohort study, ECMO-treated patients demonstrated a higher incidence of new mental health diagnoses compared with non-ECMO intensive care unit (ICU) patients (22.1 vs. 14.5 cases per 100 person-years), with a significantly increased hazard ratio for any psychiatric disorder (HR 1.24; 95% CI: 1.01–1.52) [40].

The most commonly reported conditions include depression, anxiety, and post-traumatic stress disorder (PTSD). A 2024 systematic review and meta-analysis reported pooled prevalence rates of PTSD of 19% among adult ECMO survivors and 25% among family members, while depression was identified in 24% of survivors and up to 50% of family members [41].

Further evidence from a prospective multicenter French study of patients treated with ECMO for severe COVID-19–related acute respiratory distress syndrome (ARDS) revealed that, one year after ICU admission, 44% of patients experienced clinically significant anxiety, 42% reported depressive symptoms, and 42% were at risk for PTSD. Notably, only 38% of patients returned to work after one year [42]. These findings are consistent with the broader literature on post-intensive care syndrome (PICS), which demonstrates the need for long-term psychological monitoring in this population.

Qualitative data further illustrate the depth of this burden. A recent phenomenological study of ECMO survivors identified key themes related to post-discharge experiences, including challenges in daily living, emotional distress and support needs, and unmet expectations. Overall, psychological distress and difficulties with social reintegration were found to be nearly universal among survivors [43].

### 5.3. Complex Heart Failure Surgical Procedures

Patients with advanced heart failure (HF) undergoing complex surgical interventions—including ventricular reconstruction, hybrid procedures, and high-risk coronary revascularization—share many of the psychological vulnerabilities observed in LVAD and heart transplant populations. These include preoperative anxiety, postoperative psychological distress, and the ongoing burden of advanced disease. However, this subgroup has been comparatively underexplored in the literature relative to LVAD and transplant cohorts [44].

Studies examining psychiatric comorbidity across advanced cardiac therapy populations have highlighted important differences in psychosocial profiles and therapeutic pathways. In particular, comparisons between patients receiving ventricular assist devices (VADs) and those undergoing cardiac transplantation suggest distinct patterns of psychological risk and support needs. These findings demonstrate the importance of comprehensive preoperative psychosocial evaluation and tailored postoperative support strategies for different patient groups [31].

### 5.4. Comparison with the LVAD Population

Across populations undergoing advanced cardiac surgical therapies, several common psychological themes emerge, including preoperative depression, anxiety, and overall psychological distress. These conditions may be further exacerbated during the perioperative period. Although some patients experience improvement following physical recovery, a significant burden of long-term psychological morbidity often persists.

Despite these shared features, important distinctions exist within the LVAD population. In particular, patients—especially those receiving destination therapy (DT)—face permanent dependence on a life-sustaining device. The continuous presence of the device and its associated alarms reinforces an ongoing awareness of illness and vulnerability. Moreover, device disconnection introduces a unique and potentially lethal means of self-harm that is not present in other cardiac therapy populations [45].

In contrast to transplant recipients, who may experience a sense of a “new beginning,” LVAD patients often lack this narrative, particularly in the context of lifelong device support. These differences have important implications for clinical practice, as risk assessment, screening strategies, and therapeutic interventions must be tailored to the specific challenges associated with each treatment modality.

## 6. Risk Factors for Depression and Suicidality

### 6.1. Patient-Related Factors

The risk of depression and suicidal ideation in LVAD recipients is influenced by a range of patient-related factors. Pre-existing psychiatric conditions, particularly depression, have been associated with poorer postoperative outcomes and may negatively affect adaptation to device therapy. In addition, psychosocial factors such as limited social support, social isolation, and impaired interpersonal relationships further increase vulnerability to psychological distress [18].

Data from the ASSIST-ICD study indicate that LVAD recipients who lived alone or had limited social support were overrepresented among those who attempted or completed suicide, highlighting the critical role of social isolation in suicidality within this population [29]. Personality traits associated with maladaptive coping—such as low psychological flexibility, ineffective coping strategies, and high levels of neuroticism—may further impair adjustment to long-term device dependence [18].

Younger age may also represent a risk factor, potentially reflecting a greater perceived loss of life opportunities and disruption of social roles. In patients with major depressive disorder, younger age has been associated with increased suicidal ideation [46], and a similar pattern may be relevant in younger LVAD recipients.

Neurological complications, including cerebral embolism and pump-related hemodynamic alterations, may contribute to cognitive impairment. Even transient cognitive dysfunction can adversely affect decision-making capacity and the ability to manage complex self-care requirements associated with LVAD therapy.

### 6.2. Disease-Related Factors

The severity and chronicity of advanced heart failure (HF) independently contribute to the risk of depression. The cumulative burden of illness—characterized by persistent dyspnea, fatigue, pain, and functional limitations—is inherently demoralizing. The progressive inability to perform previously routine activities can undermine self-efficacy, autonomy, and personal identity.

Recurrent hospitalizations related to clinical deterioration or device complications further disrupt social and occupational functioning, increase financial stress, and contribute to a persistent sense of loss of control [47]. In addition, neurological complications, including stroke, occur in approximately 10–15% of LVAD recipients and may precipitate or exacerbate depressive symptoms through both direct neurological injury and the resulting functional impairment [48].

Furthermore, the requirement for lifelong anticoagulation imposes additional limitations on daily activities, contributes to anxiety, and increases the risk of bleeding complications, thereby adding to the overall psychological burden [49].

### 6.3. Device- and Surgery-Related Factors

LVAD therapy introduces a unique set of device- and surgery-related psychological stressors. Device alarms—triggered by low battery, controller malfunction, or hemodynamic disturbances—are often frequent and unpredictable, contributing to a persistent state of vigilance and psychological hyperarousal. Living with a life-sustaining device also entails a constant awareness of vulnerability, including the risk of accidental or intentional disconnection.

The driveline, which connects the implanted pump to the external controller, requires continuous care to prevent infection and mechanical complications. Its visible and external nature serves as a constant reminder of technological dependence and may negatively influence body image and self-perception [33].

In addition, major postoperative complications—such as right heart failure, bleeding, infection, and neurological events—contribute to increased physical morbidity and may attenuate the initial optimism associated with LVAD implantation. These complications can precipitate or exacerbate depressive symptoms, particularly when recovery is prolonged or outcomes are uncertain [35,50].

### 6.4. Psychosocial and Caregiver Factors

The caregiver relationship is a central determinant of psychological outcomes in patients undergoing LVAD therapy. Most LVAD programs require the presence of a dedicated caregiver, typically a spouse or close family member, who must be trained in device management, emergency procedures, and driveline care. This role is highly demanding, both in practical and emotional terms.

A mixed-methods longitudinal study by McIlvennan et al. [13] demonstrated that increased caregiver stress is independently associated with lower caregiver preparedness, higher levels of caregiver depressive symptoms, and reduced patient quality of life. These findings suggest the presence of a reciprocal and reinforcing cycle of distress within the patient–caregiver dyad. Caregiver burden is often most pronounced within the first one to three months following LVAD implantation, and multiple studies summarized by Streur et al. [51] report that a substantial proportion of caregivers experience clinically significant levels of strain during this period.

In addition, financial stress—including healthcare costs, loss of employment, and reduced income due to long-term disability—further contributes to the psychosocial burden experienced by both patients and their families. Barriers to social reintegration, such as limited ability to return to work, travel restrictions, and the visibility of medical devices, may further restrict participation in activities that support psychological well-being and social identity [52].

**Figure 3:**
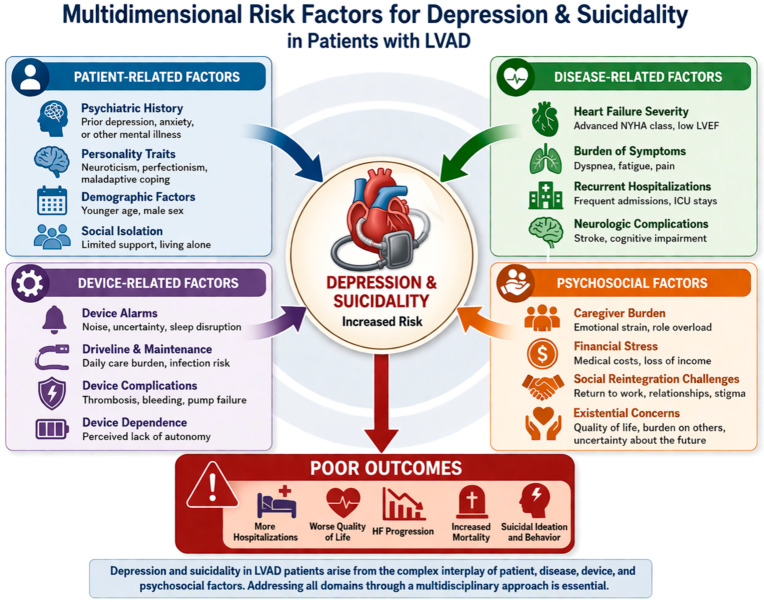
Multidimensional risk factors for depression and suicidality in patients with left ventricular assist devices (LVADs). Depression and suicidal ideation in LVAD recipients arise from a complex and interacting set of risk factors across four major domains. Patient-related factors include pre-existing psychiatric illness, maladaptive personality traits, younger age, and social isolation. Disease-related factors encompass advanced heart failure severity, symptom burden, recurrent hospitalizations, and neurologic complications. Device-related factors involve the psychological and physical burden of device dependence, including alarms, driveline care, complications, and body image disturbance. Psychosocial factors include caregiver burden, financial stress, impaired social reintegration, and existential distress. These domains interact dynamically to increase vulnerability to depression and suicidality, ultimately contributing to poorer clinical outcomes, including reduced quality of life, increased hospitalizations, and higher mortality. Recognition of this multidimensional risk profile supports the need for comprehensive, multidisciplinary assessment and intervention. This figure represents a conceptual schematic model and does not depict primary data.

The multidimensional interplay of patient-, disease-, device-, and psychosocial-related risk factors contributing to depression and suicidality in LVAD patients is summarized in Figure 3.

## 7. Pathophysiological and Psychosocial Mechanisms

### 7.1. Biological Mechanisms

The relationship between LVAD support and depression is mediated by multiple interrelated biological pathways. Both advanced heart failure (HF) and device therapy exert direct physiological effects, while additional hemodynamic and hematological mechanisms further contribute to neuropsychiatric vulnerability. In advanced HF, sustained activation of the hypothalamic–pituitary–adrenal (HPA) axis and the sympathetic nervous system has been well documented [53].

This chronic activation leads to elevated circulating levels of cortisol and catecholamines, which in turn disrupt central monoaminergic neurotransmitter systems, including serotonin, norepinephrine, and dopamine—key regulators of mood and emotional processing [53,54,55].

Neuroinflammation represents another critical pathway. The ischemic, hemodynamic, and metabolic disturbances associated with HF promote increased levels of pro-inflammatory cytokines, such as interleukin-6 (IL-6) and tumor necrosis factor-α (TNF-α). These cytokines can cross the blood–brain barrier and activate neuroinflammatory cascades, resulting in impaired neuronal function, reduced neuroplasticity, and the development of depressive symptoms [56,57].

The heart–brain axis—representing the bidirectional autonomic, endocrine, and inflammatory communication between the cardiovascular and central nervous systems—is profoundly disrupted in advanced HF. Cardiac dysfunction generates abnormal interoceptive signals that alter emotional regulation and contribute to mood disorders [57]. Within the specific context of LVAD support, additional mechanisms may further influence this axis.

Modern LVADs generate continuous, non-pulsatile blood flow, which may alter cerebral perfusion dynamics and microvascular function, potentially contributing to neuropsychiatric manifestations, including depression. In addition, acquired von Willebrand factor deficiency and the associated coagulopathic state may predispose to microvascular cerebral ischemia. Endothelial dysfunction and device-related inflammatory responses may further amplify HF-related neuroinflammatory processes [23].

These observations are consistent with broader evidence linking mental stress to cardiovascular disease through interconnected neuroendocrine, inflammatory, and autonomic pathways. A recent narrative review by Gill et al. highlighted the role of chronic psychological stress in activating the hypothalamic–pituitary–adrenal (HPA) axis, increasing sympathetic nervous system activity, and promoting systemic inflammation, all of which contribute to both cardiovascular dysfunction and the development of depressive symptoms [58]. Importantly, these mechanisms provide a unifying biological framework that connects emotional stress, cardiac disease progression, and mental health outcomes. In the context of advanced heart failure and LVAD support, these stress-related pathways may be further amplified by disease severity and device-related physiological alterations, reinforcing the close interaction between psychological and cardiovascular processes [58,59].

Collectively, these interrelated mechanisms—including neurohormonal dysregulation, systemic and neuroinflammation, and altered hemodynamics—create a neurobiological environment that increases susceptibility to clinically significant depression in LVAD recipients.

### 7.2. Psychological Mechanisms

In addition to biological factors, a range of psychological mechanisms contributes to the development of depression and suicidality in LVAD patients. A central component is the disruption of perceived control, as device dependence imposes significant limitations on autonomy. This loss of control can foster feelings of helplessness and hopelessness, both of which are strongly associated with increased suicide risk [60,61].

Patients with advanced cardiac disease frequently experience existential distress as they confront issues related to mortality, dependency, and a fundamentally altered sense of self. For individuals receiving LVAD therapy as destination therapy (DT), the recognition that the device represents a life-sustaining but non-curative intervention may further intensify this distress. This awareness can trigger grief-related responses associated with the loss of a previously anticipated future and identity [32,61].

Psychological pain has also been identified as a key construct linking emotional distress to suicidality. Defined as the subjective experience of intense and intolerable inner suffering arising from unmet psychological needs, psychological pain has been consistently associated with suicidal ideation and behavior in both clinical and non-clinical populations [62]. In patients with advanced HF, this form of distress is likely to be particularly pronounced due to the cumulative burden of illness and treatment [61,62].

Following LVAD implantation, many patients experience an initial period of physical improvement—often referred to as the “honeymoon phase”—characterized by symptom relief and psychological optimism. However, this phase is frequently followed by a period of psychological recalibration, during which patients come to terms with the long-term constraints of device dependence. This transition represents a critical period of vulnerability, during which the risk of depression and psychological distress may increase.

### 7.3. Social and Environmental Factors

In patients undergoing advanced cardiac therapies, mental health outcomes are shaped not only by biological and psychological mechanisms but also by social and environmental factors.

Reduced social participation, driven by physical limitations and lifestyle restrictions associated with device therapy, can significantly diminish opportunities for social engagement and role fulfillment—both of which are important protective factors against depression. Social isolation related to chronic and severe illness, combined with loss of employment, financial strain, and disruption of family dynamics, further amplifies psychosocial burden.

Environmental stressors may also play a critical role in triggering acute psychological crises. The case reported by Jiménez-Blanco Bravo et al. [30], describing a suicide attempt in an LVAD patient without prior psychiatric history during COVID-19–related lockdown, highlights the potential impact of social isolation as an independent risk factor. The COVID-19 pandemic has thus provided a natural context for understanding the additive effects of environmental stressors on psychological vulnerability in LVAD populations.

**Figure 4:**
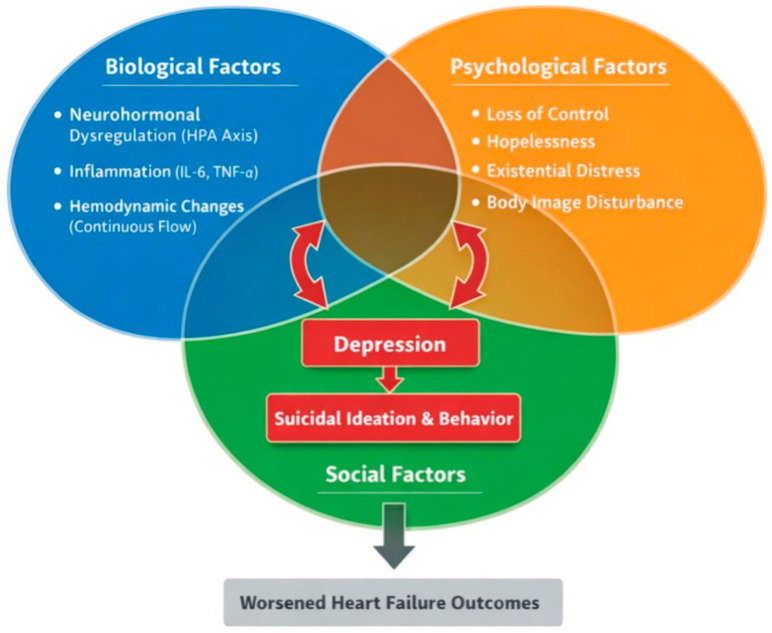
Biopsychosocial integrative model of depression and suicidality in LVAD patients. This figure represents a conceptual schematic model and does not depict primary data.

To further clarify the dynamic interplay between these domains, it is important to emphasize that the relationships within the biopsychosocial model are bidirectional and mutually reinforcing. For example, biological factors such as neuroinflammation and neurohormonal dysregulation may increase vulnerability to psychological distress, while persistent depressive symptoms can, in turn, exacerbate physiological stress responses and inflammatory pathways. Similarly, social isolation and caregiver burden may intensify psychological distress, which can lead to reduced treatment adherence and poorer clinical outcomes, thereby worsening underlying disease status. Conversely, supportive social environments and effective coping strategies may mitigate both psychological and biological risk. These interactions are not linear but evolve over time, highlighting the need for continuous, integrative assessment across all domains. The interplay of these biological, psychological, and social mechanisms is summarized in Figure 4.

Taken together, these findings support a unified biopsychosocial model of depression and suicidality in patients with advanced heart failure and LVAD support. At the biological level, heart failure itself is associated with neurohormonal activation, systemic inflammation, and alterations in the heart–brain axis, all of which contribute to a neurobiological vulnerability to depression. LVAD-specific factors may further modulate these pathways through continuous-flow hemodynamics, altered cerebral perfusion, and device-related inflammatory responses, potentially amplifying underlying susceptibility.

At the psychological level, patients with advanced heart failure frequently experience loss of autonomy, uncertainty regarding prognosis, and existential distress. In LVAD recipients, these processes are intensified by long-term device dependence, altered body image, and the constant awareness of technological support, which may reinforce feelings of vulnerability and loss of control.

Social and environmental factors—including caregiver burden, social isolation, and reduced participation in daily activities—interact with both biological and psychological processes to further increase the risk of depression and suicidality. Importantly, these domains do not operate independently but are dynamically interconnected. Biological vulnerability may lower resilience to psychosocial stressors, while psychological distress and social isolation may, in turn, exacerbate neuroinflammatory and neuroendocrine dysregulation.

Within this framework, suicidality can be understood as the result of cumulative and interacting risk across these domains, with LVAD-specific factors introducing an additional layer through access to device-related means of self-harm. This integrative model highlights the need to conceptualize depression and suicidality in LVAD patients not as isolated phenomena, but as the outcome of complex, interacting systems that evolve over time.

## 8. Screening and Assessment

### 8.1. Preoperative Psychiatric Evaluation

Preoperative psychiatric evaluation is an essential component of the assessment process for LVAD candidacy and heart transplant listing, as recommended by current clinical guidelines [63,64]. The Stanford Integrated Psychosocial Assessment for Transplantation (SIPAT) has emerged as one of the most widely used standardized instruments for this purpose. SIPAT evaluates patients across four key domains: readiness for transplantation, social support system, psychological stability and psychopathology, and lifestyle factors including substance use [64,65].

Higher SIPAT scores have been associated with adverse post-implant outcomes, including increased rates of emergency department visits, urgent care utilization, and hospital readmissions following LVAD implantation. Olt et al. [64] further demonstrated that the SIPAT scoring system places particular emphasis on psychological stability and psychopathology, demonstrating their importance in predicting unfavorable clinical outcomes.

Importantly, psychosocial evaluation should not be viewed solely as a gatekeeping tool for determining treatment eligibility. Rather, it should serve as a means of identifying modifiable risk factors that can be addressed through targeted pre-implant interventions. Notably, inadequate social support is often a more significant contraindication to advanced therapies than the mere presence of a psychiatric history.

### 8.2. Screening Tools for Depression

Several validated instruments are available for the screening of depression in patients with cardiovascular disease, each with characteristics that may make them more suitable for specific clinical settings. The Patient Health Questionnaire-9 (PHQ-9) is among the most widely used and guideline-supported tools for depression screening.

The American Heart Association recommends routine depression screening in cardiovascular inpatients. A PHQ-9 score of ≥10 is commonly used as the clinical threshold for probable major depression, providing an optimal balance between sensitivity and specificity [66].

The Hospital Anxiety and Depression Scale (HADS) offers the advantage of simultaneously assessing both anxiety and depression and has demonstrated good diagnostic performance in cardiac populations. The Beck Depression Inventory-II (BDI-II) provides a more comprehensive evaluation of depression severity and has been extensively utilized in LVAD studies as both a screening instrument and an outcome measure [24,67].

It is important to note that the use of non-cardiac-specific instruments may introduce diagnostic challenges, as somatic symptoms such as fatigue, sleep disturbance, and impaired concentration—common in HF—may overlap with depressive symptoms and lead to overestimation of depression severity. Therefore, screening results should always be interpreted within the broader clinical context [66].

### 8.3. Screening for Suicidality

Current psychiatric and cardiovascular guidelines recommend direct and routine inquiry about suicidal ideation as part of comprehensive clinical assessment. This evaluation should be incorporated both into the pre-implant psychiatric assessment and into ongoing psychological follow-up after LVAD implantation. Validated instruments such as the Columbia Suicide Severity Rating Scale (C-SSRS) and the Ask Suicide-Screening Questions (ASQ) toolkit provide structured and feasible approaches for assessing suicide risk in clinical settings [68,69,70].

In LVAD populations, suicidality assessment should extend beyond standard evaluation of intent and planning to include careful exploration of device-specific considerations, such as thoughts related to device disconnection. These discussions must be conducted in a clear, sensitive, and compassionate manner, ensuring appropriate communication without promoting harmful detail.

Evidence from the ASSIST-ICD study highlights the importance of systematic screening. Among LVAD recipients who attempted or completed suicide, only a minority had a prior psychiatric diagnosis, while the majority had previously expressed symptoms such as sadness or hopelessness. These findings suggest that ongoing, structured screening programs may facilitate early identification of at-risk individuals before progression to crisis [29].

Commonly used and validated tools for screening depression and suicidality in advanced cardiac populations are summarized in Table 2.

Screening tools should be interpreted in the clinical context of advanced heart failure and LVAD support, where somatic symptoms may overlap with depressive features. Positive screening results should prompt comprehensive psychiatric evaluation rather than serve as standalone diagnostic criteria.

### 8.4. Role of Multidisciplinary Teams

Effective mental health surveillance in patients undergoing advanced cardiac therapies requires a coordinated, multidisciplinary approach. The 2025 European Society of Cardiology (ESC) Clinical Consensus Statement on Mental Health and Cardiovascular Disease emphasizes the integration of mental health into personalized cardiovascular care and advocates for the development of innovative, multidisciplinary “psycho-cardiology” care models [14].

An optimal LVAD care team should include cardiologists, cardiac surgeons, dedicated LVAD coordinators, clinical psychologists or psychiatrists, palliative care specialists, and social workers. Evidence from the ASSIST-ICD study highlights the potential protective role of structured team-based care. In this cohort, 60.5% of patients who did not attempt suicide were followed in centers with LVAD coordinator involvement, compared with only 20% of those who attempted or completed suicide [29].

LVAD coordinators, who maintain continuous and close contact with patients throughout long-term follow-up, are uniquely positioned to identify early signs of psychological distress. Regular psychological assessment within this framework may serve as an accessible and cost-effective first-line screening strategy for mental health concerns [71].

## 9. Management Strategies

### 9.1. Pharmacological Treatment

Selective serotonin reuptake inhibitors (SSRIs) are the most commonly prescribed antidepressants in cardiac populations [72] and are generally considered the first-line pharmacological treatment for depression in patients with heart failure (HF) and LVAD support. SSRIs have a favorable cardiovascular safety profile, with minimal effects on heart rate variability, blood pressure, or left ventricular ejection fraction, and may exert mild antiplatelet effects [72,73,74]. Large randomized trials in cardiac populations, such as the SADHART-CHF study, have further supported the relative cardiovascular safety of SSRIs, although their impact on hard clinical outcomes remains less clear [75].

However, the impact of SSRI-induced platelet serotonin depletion must be carefully considered in LVAD patients, who are already at increased risk of bleeding due to mandatory long-term anticoagulation. In a 2022 study of 92 patients with ventricular assist devices, the use of serotonergic reuptake inhibitors was associated with a significantly increased bleeding risk (z = 2.091, *p* = 0.037) [73]. Similarly, a 2024 nested cohort study demonstrated that concomitant use of SSRIs and oral anticoagulants was associated with a 33% increased risk of major bleeding, with the highest risk observed during the first month of combined therapy [74]. These findings are consistent with broader pharmacovigilance data demonstrating increased gastrointestinal and intracranial bleeding risk with SSRIs, particularly when combined with antithrombotic agents [76].

Given that LVAD recipients require lifelong anticoagulation, careful individualized assessment of the risk–benefit balance is essential when initiating antidepressant therapy. Close clinical monitoring, particularly during treatment initiation, and clear communication with patients regarding potential risks are critical. Dose selection, gradual titration, and interdisciplinary coordination between cardiology and psychiatry are essential components of safe pharmacological management.

Beyond SSRIs, serotonin–norepinephrine reuptake inhibitors (SNRIs), including venlafaxine and duloxetine, represent an important therapeutic alternative, particularly in patients with comorbid anxiety or chronic pain syndromes. However, SNRIs may increase blood pressure and heart rate through noradrenergic effects, which may be problematic in patients with advanced HF or hemodynamic instability. Evidence suggests that venlafaxine is associated with dose-dependent increases in blood pressure and requires careful monitoring in cardiovascular populations [77]. Therefore, SNRIs should be used cautiously and preferably in stable patients with close hemodynamic surveillance.

Tetracyclic antidepressants, particularly mirtazapine, may offer specific advantages in LVAD populations. Mirtazapine is associated with sedative, anxiolytic, and appetite-stimulating properties, which may be beneficial in patients with insomnia, cachexia, or poor nutritional status—common features in advanced HF. Importantly, mirtazapine has minimal effects on cardiac conduction and does not significantly inhibit platelet aggregation, potentially making it a safer alternative in patients at high bleeding risk [78]. However, its use must be balanced against potential adverse effects, including weight gain and sedation.

Multimodal antidepressants such as vortioxetine represent a newer class of agents with a unique mechanism of action involving serotonin transporter inhibition and receptor modulation. Vortioxetine has demonstrated efficacy not only in improving depressive symptoms but also in enhancing cognitive function, which may be particularly relevant in patients with HF who frequently exhibit cognitive impairment [79]. Importantly, available evidence suggests that vortioxetine has a favorable cardiovascular safety profile, with minimal effects on QT interval, heart rate, or blood pressure [80]. Although specific data in LVAD populations are lacking, its pharmacological profile makes it a promising option in selected patients.

Other agents, such as bupropion, may also be considered in individualized cases. Bupropion, a norepinephrine–dopamine reuptake inhibitor, does not significantly affect platelet function and therefore does not appear to increase bleeding risk. However, its potential to elevate blood pressure and its contraindication in patients with seizure risk must be taken into account when prescribing in cardiovascular populations.

Tricyclic antidepressants are generally avoided in patients with cardiovascular disease due to their potential to induce arrhythmias. In cases of acute suicidality, pharmacological treatment should be complemented by close psychiatric monitoring and appropriate safety planning. Their anticholinergic effects, risk of QT prolongation, and association with orthostatic hypotension further limit their use in patients with advanced HF.

Overall, pharmacological treatment of depression in LVAD patients requires a highly individualized and multidisciplinary approach. Selection of antidepressant therapy should incorporate cardiovascular safety, bleeding risk, comorbidities, and patient preferences. Given the complexity of these patients, ongoing monitoring and close collaboration between cardiology, psychiatry, and primary care teams are essential to optimize both psychological and clinical outcomes.

### 9.2. Psychotherapy

Psychotherapy is both effective and feasible in cardiac populations and represents a key evidence-based treatment for depression. Cognitive behavioral therapy (CBT) has the strongest evidence base and has been extensively studied in patients with heart failure (HF). A 2023 meta-analysis of randomized controlled trials demonstrated that CBT produced significant long-term improvements (four to nine months) in both anxiety and depression compared with non-CBT interventions, although short-term effects (within three months) were less consistent [81].

Combined interventions appear to offer additional benefit. In patients with NYHA Class II–III HF, CBT delivered alongside exercise-based cardiac rehabilitation was more effective than either intervention alone, particularly among individuals with moderate-to-severe depression [82,83].

Acceptance and Commitment Therapy (ACT) represents a newer therapeutic approach that may be particularly well suited to LVAD populations. ACT focuses on enhancing psychological flexibility by encouraging acceptance of unchangeable circumstances and promoting value-driven behavior, making it conceptually aligned with the challenges of chronic device dependence. A 2025 systematic review by Grimaldi et al. reported preliminary evidence that ACT-based interventions may improve coping, enhance self-regulation, and reduce psychological distress in patients with HF and other cardiovascular diseases, although the current evidence base is limited by small sample sizes and methodological heterogeneity [84].

Other psychotherapeutic approaches, including supportive psychotherapy, motivational interviewing, and problem-solving therapy, may also be beneficial, particularly for patients with limited insight into their psychological distress or those who are less receptive to structured therapeutic modalities.

### 9.3. Multidisciplinary Care Models and Palliative Care Integration

Patients with LVAD support benefit from care models that integrate psychosocial support throughout the entire continuum of care. Multidisciplinary approaches that incorporate mental health and supportive services are particularly important in addressing the complex and evolving needs of this population. Despite these needs, specialist palliative care (SPC) remains underutilized, even though early integration is recommended within LVAD programs.

The 2024 multicenter European study by Tenge et al. [85] represents one of the most comprehensive evaluations of SPC involvement in LVAD patients. Although approximately two-thirds of LVAD centers reported access to SPC services, initial contact with palliative care teams most frequently occurred late in the disease trajectory, often shortly before death in the intensive care unit. This finding highlights a pattern of delayed integration [85].

Importantly, palliative care teams consistently identified a higher symptom burden than referring clinicians. Commonly reported symptoms included anxiety, weakness, dyspnea, and caregiver strain, suggesting that psychological and supportive care needs are often underrecognized by primary LVAD teams [86].

Early integration of palliative care offers several benefits, including the facilitation of advance care planning, support in setting realistic expectations, reduction in caregiver burden, and provision of a structured framework for end-of-life discussions, including preferences regarding device deactivation [85].

### 9.4. Psychosocial Support and Digital Innovations

Effective management of depression and suicidality in LVAD patients requires comprehensive psychosocial support, including patient education, peer support programs, caregiver training, and access to community resources. Peer support—defined as connecting LVAD recipients with individuals who have undergone similar experiences—has been shown to enhance coping self-efficacy while reducing stigma and social isolation [52].

Interventions targeting caregivers are equally essential. Given the bidirectional nature of psychological distress within the patient–caregiver dyad, patient-focused interventions that do not also address caregiver needs are unlikely to be fully effective [13].

Emerging digital mental health approaches offer promising opportunities to expand access to care, particularly for patients with limited mobility or barriers to in-person services. Telehealth-delivered psychiatric care has demonstrated significant effectiveness; in a large real-world cohort, patients receiving telepsychiatry for suicidal ideation had 4.3-fold higher odds of remission compared with controls [87].

In addition, digital platforms incorporating ecological momentary assessment, app-based symptom monitoring, and algorithm-driven decision support may enable proactive and continuous mental health surveillance. These innovations have the potential to address the current gaps in care, which are often characterized by fragmented and episodic psychological support.

**Figure 5:**
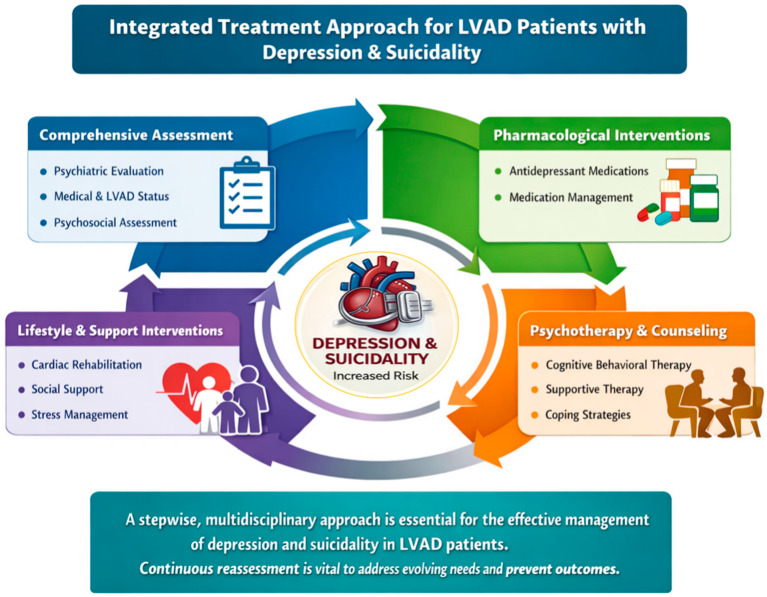
Integrated clinical approach for the assessment and management of depression and suicidality in patients with left ventricular assist devices (LVADs). Management of depression and suicidality in LVAD patients requires a structured, multidisciplinary, and continuous care model. Initial comprehensive assessment includes psychiatric evaluation, medical and device-related status, and psychosocial context. Treatment strategies encompass pharmacological interventions (e.g., antidepressant therapy with careful consideration of bleeding risk), evidence-based psychotherapies such as cognitive behavioral therapy, and supportive interventions including cardiac rehabilitation, social support, and stress management. Given the dynamic nature of psychological distress in this population, continuous reassessment and treatment adjustment are essential. Integration of cardiology, psychiatry, psychology, and social care is critical to optimize outcomes and reduce the burden of depression and suicidal behavior. This figure represents a conceptual schematic model and does not depict primary data.

A structured, multidisciplinary approach to the assessment and management of depression and suicidality in LVAD patients is illustrated in Figure 5. Key management strategies are summarized in Table 3.

Management of depression and suicidality in LVAD patients should be individualized and continuously adapted over time. Close monitoring is essential, particularly when initiating pharmacological therapy in anticoagulated patients and when assessing suicide risk in the context of device access.

### 9.5. Integrated Clinical Management Framework

Based on the available evidence, a structured and longitudinal clinical pathway can be proposed for the assessment and management of depression and suicidality in patients undergoing LVAD implantation and other advanced cardiac therapies. This framework integrates preoperative evaluation, postoperative monitoring, risk stratification, and targeted intervention strategies across the continuum of care.

#### 9.5.1. Preoperative Phase

Comprehensive psychosocial and psychiatric evaluation should be performed prior to LVAD implantation, including assessment of depression, prior psychiatric history, substance use, social support, and caregiver availability. Standardized tools such as the SIPAT may assist in identifying high-risk patients and informing individualized care planning [64,65].

#### 9.5.2. Early Postoperative Phase

Following implantation, patients should undergo early reassessment of psychological status, as this period is characterized by significant physical recovery and psychological adjustment. Screening for depression and suicidal ideation should be systematically performed using validated instruments (e.g., PHQ-9, C-SSRS), with particular attention to acute stressors, complications, and changes in functional status [23,66].

#### 9.5.3. Long-Term Follow-Up and Risk Stratification

Psychological monitoring should be continuous and longitudinal, recognizing that depression and suicidality may evolve over time. Patients may be stratified into low-, moderate-, and high-risk categories based on clinical factors, including prior psychiatric history, social isolation, caregiver burden, device-related complications, and expressed psychological distress. This stratification may guide the intensity of follow-up and intervention.

#### 9.5.4. Intervention Strategies

Management should be multidisciplinary and tailored to patient needs. Pharmacological treatment, psychotherapeutic interventions, and psychosocial support should be integrated within routine care. In high-risk patients, closer psychiatric follow-up, safety planning, and coordinated care involving mental health specialists are required.

#### 9.5.5. Advanced Care and Ethical Considerations

In patients with persistent psychological distress or advanced disease, integration of palliative care is essential. Discussions regarding goals of care, quality of life, and, when appropriate, device deactivation should be conducted within a structured ethical framework and with multidisciplinary input [85,87].

#### 9.5.6. Digital and Supportive Strategies

Telemedicine and digital mental health tools may support continuous monitoring and improve access to care, particularly in patients with limited mobility or barriers to in-person follow-up [14].

This integrated framework highlights the need for a proactive, continuous, and multidisciplinary approach, emphasizing that psychological care should be embedded within standard LVAD management rather than applied reactively.

To facilitate translation of this framework into routine clinical practice, several pragmatic considerations may be highlighted. In particular, mental health screening may be incorporated at predefined stages of care, including the pre-implant evaluation, early post-discharge period, and during longitudinal follow-up. The use of brief, validated instruments such as the PHQ-9 may support efficient assessment in busy clinical settings, while the inclusion of simple, structured questions regarding suicidal ideation can enhance early detection of psychological distress. Establishing clear referral pathways to mental health professionals within LVAD programs is essential to ensure timely access to specialized care. In addition, regular multidisciplinary discussions and active involvement of caregivers in education and follow-up may further support adherence, early risk identification, and overall psychosocial well-being. Collectively, these measures may help us to operationalize the proposed framework in a feasible and clinically meaningful manner.

## 10. Ethical Considerations

### 10.1. Decision-Making Capacity and Pre-Implant Evaluation

The decision to proceed with implantation of a life-sustaining device such as an LVAD raises fundamental ethical questions regarding informed consent and decision-making capacity. These issues must be carefully addressed during the pre-implant evaluation process [88,89].

Patients with significant depression and/or cognitive impairment may have a reduced ability to make fully informed and autonomous decisions regarding LVAD implantation. Therefore, assessment of decision-making capacity should be systematically performed, clearly documented, and, where possible, optimized through appropriate treatment prior to proceeding with implantation.

Pre-implant psychiatric evaluation should extend beyond risk stratification to include a thorough assessment of the patient’s understanding of the procedure, its long-term implications, and the possibility of future device deactivation. Equally important is the evaluation and support of patient agency, ensuring that individuals are able to meaningfully engage with information and participate in shared decision-making [89,90,91].

### 10.2. LVAD Deactivation and End-of-Life Care

LVAD deactivation presents significant ethical complexity and may generate substantial moral distress among clinical teams, particularly when undertaken at the patient’s request or as part of end-of-life care planning. However, LVAD deactivation is widely considered ethically permissible and is generally regarded as analogous to the withdrawal of other life-sustaining therapies, such as mechanical ventilation. It is not classified as physician-assisted death, and the cause of death is attributed to the underlying advanced heart failure [92].

In a 2025 vignette-based study, Fried et al. [93] found that clinicians perceived LVAD deactivation as more ethically complex than discontinuation of hemodialysis. Moreover, clinicians were less likely to honor patient requests for LVAD deactivation across multiple clinical scenarios, highlighting the persistence of ethical uncertainty and moral distress in clinical practice.

A proportionality-based framework—taking into account the anticipated benefits of continued therapy, the patient’s values and preferences, and the overall burden of ongoing device support—provides a structured and ethically grounded approach to decision-making regarding deactivation [94]. Ensuring alignment with patient preferences, alongside comprehensive symptom management and, when appropriate, palliative sedation, is essential to support patient comfort and dignity at the end of life.

### 10.3. Suicide Risk and Device Access

One of the most ethically distinctive aspects of suicidality in LVAD patients is the potential for device disconnection, which provides direct access to a lethal means. This reality introduces additional responsibilities for clinical teams beyond standard approaches to suicide risk management.

Preoperative counseling should include clear and sensitive discussions regarding the life-sustaining function of the device, as well as assessment of suicide risk and expectations for ongoing psychological support. Suicide risk evaluation and safety planning should be explicitly incorporated into longitudinal follow-up. At the same time, communication must be carefully framed to avoid inadvertently providing actionable information that could facilitate self-harm [95].

A central ethical challenge lies in balancing respect for patient autonomy—including the right to refuse or withdraw life-sustaining treatment—with the duty to prevent harm. Distressed LVAD patients should be managed through a coordinated, multidisciplinary approach involving ethics consultation, psychiatry, and palliative care services to support complex decision-making and ensure patient-centered care.

**Figure 6:**
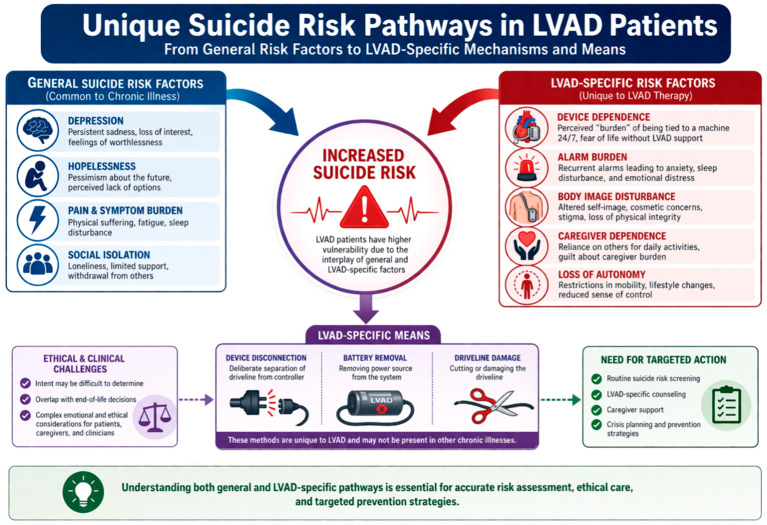
Suicide risk pathways unique to patients with left ventricular assist devices (LVADs). Suicide risk in LVAD recipients arises from the interaction between general risk factors common to chronic illness and factors specific to device-supported life. General contributors include depression, hopelessness, physical symptom burden, and social isolation. LVAD-specific factors include device dependence, alarm-related distress, body image disturbance, caregiver reliance, and loss of autonomy. Importantly, LVAD patients have access to unique means of self-harm, including device disconnection, battery removal, and driveline damage, which are not present in other chronic disease populations. These features introduce additional ethical and clinical challenges related to intent assessment, end-of-life decision-making, and patient autonomy. Recognition of these distinct pathways is essential for targeted screening, risk mitigation, and the development of tailored preventive strategies in this vulnerable population. This figure represents a conceptual schematic model and does not depict primary data.

The unique pathways and mechanisms contributing to suicide risk in LVAD patients, including device-specific factors and access to lethal means, are illustrated in Figure 6.

Beyond its clinical implications, the availability of device-specific means of self-harm in LVAD recipients represents a fundamentally distinct dimension of suicidality compared with other chronic diseases. In most medical conditions, suicidal behavior typically involves external methods that are not inherently linked to the treatment itself. In contrast, LVAD patients live with continuous access to a life-sustaining device, the manipulation or discontinuation of which can directly result in death [23,29]. This creates a unique convergence between therapeutic technology and potential means of self-harm, blurring the boundaries between medical management, patient autonomy, and suicide risk.

This phenomenon has important implications for clinical practice. Patient education must extend beyond technical device management to include careful, ethically appropriate discussions about the life-sustaining nature of the device and the importance of adherence [87]. At the same time, long-term follow-up should incorporate ongoing assessment of psychological adaptation to device dependence, as risk may evolve over time rather than being confined to the peri-implant period [23,29].

From an ethical perspective, the distinction between intentional device manipulation as an act of self-harm and patient-requested device deactivation in the context of end-of-life care introduces additional complexity. While the latter is widely considered ethically permissible when aligned with patient preferences and clinical context, distinguishing between these scenarios in practice may be challenging, particularly in patients with fluctuating decision-making capacity or untreated psychiatric symptoms [92,93,94].

Compared with other chronic disease populations, this dual role of the device—as both a life-sustaining therapy and a potential means of self-harm—necessitates a more nuanced and proactive approach to risk assessment, patient counseling, and multidisciplinary care. Recognizing this unique feature of LVAD therapy is essential for the development of tailored preventive strategies and for addressing the broader ethical and clinical challenges associated with device-dependent life [45].

## 11. Clinical Implications

The findings of this review have several important implications for clinical practice. Routine and longitudinal screening for depression should be systematically implemented in patients undergoing LVAD implantation and other advanced cardiac therapies. Validated instruments, such as the Patient Health Questionnaire-9 (PHQ-9), should be applied at predefined time points, including pre-implant evaluation, early post-implant recovery, and throughout long-term follow-up. Screening protocols should be structured, standardized, and adapted to the specific characteristics of LVAD populations [23].

Assessment of suicidal ideation should be incorporated into routine clinical care. This includes direct and structured inquiry, as well as evaluation of device-specific risks, such as potential device disconnection. Suicide risk assessment should be performed regularly during follow-up and integrated into standard clinical workflows.

Mental health professionals should be integrated as core members of advanced cardiac therapy programs. Referral pathways should be proactive and embedded within routine care, rather than limited to crisis-driven consultations. Multidisciplinary collaboration among cardiology, cardiac surgery, psychiatry or psychology, and allied health professionals should be established to ensure coordinated and comprehensive patient management.

Caregiver support should be systematically incorporated into LVAD care pathways. Structured interventions, including education, counseling, and regular assessment of caregiver burden, should be initiated during the pre-implant evaluation phase and maintained throughout follow-up, recognizing the interdependence between patient and caregiver well-being [23,60].

Early integration of palliative care should be implemented, ideally at the stage of LVAD evaluation or implantation. Palliative care involvement should focus on symptom management, support for coping strategies, and facilitation of advance care planning [85,96].

Standardized mental health training should be provided to LVAD coordinators and other non-psychiatric healthcare professionals. Given their continuous contact with patients, these team members should be equipped to identify early signs of psychological distress and initiate timely referral to specialized care [13,60].

Finally, digital mental health interventions and telepsychiatry services should be incorporated into care pathways where feasible, in order to improve access to mental health support, particularly for patients with limited mobility or barriers to in-person follow-up [14].

## 12. Discussion and Future Research Directions

Depression and suicidality are increasingly recognized as integral components of the clinical trajectory in patients with advanced heart failure (HF), particularly those supported with left ventricular assist devices (LVADs). The reported prevalence of depression in this population, ranging from approximately 15% to 42% depending on timing and assessment methodology [23], reflects not only methodological variability but also the dynamic nature of psychological adaptation across different stages of disease and device support. This variability underlines the importance of conceptualizing depression in LVAD recipients as a fluctuating and context-dependent condition rather than a static comorbidity.

Although suicidal behavior remains relatively infrequent, its occurrence at rates higher than those observed in the general population and in other chronic disease cohorts suggests a distinct risk profile associated with LVAD therapy. The findings of the ASSIST-ICD study, reporting a 2% rate of suicide attempts or completed suicide over a median follow-up of 18 months [29], highlight the clinical relevance of this issue. Importantly, the availability of device-specific means of self-harm—such as driveline disconnection or battery interruption—introduces a unique dimension of suicidality that is not present in other medical contexts [23,29]. This feature distinguishes LVAD recipients from other chronic disease populations and supports the conceptualization of suicidality in this setting as a device-mediated and context-specific phenomenon.

The pathogenesis of depression in LVAD recipients is best understood within a multidimensional biopsychosocial framework. Biological mechanisms, including neurohormonal activation, systemic inflammation, and altered cerebral perfusion, create a neurobiological substrate that may predispose to mood disorders. These processes are well established in HF and may be further modified by continuous-flow physiology and device-related factors. Emerging evidence linking systemic inflammation and endothelial dysfunction to depressive symptoms provides additional support for shared pathophysiological pathways between cardiovascular and psychiatric disease [97]. At the same time, psychological factors—such as loss of autonomy, altered body image, and existential distress—interact with social determinants, including caregiver burden and social isolation, to shape individual vulnerability. This integrated perspective aligns with broader literature demonstrating that depression both contributes to and is exacerbated by cardiovascular disease through behavioral and biological mechanisms [98].

When compared with other advanced cardiac therapies, including heart transplantation and extracorporeal membrane oxygenation (ECMO), LVAD therapy appears to confer a distinct psychosocial profile. Unlike transplant recipients, who may experience a sense of therapeutic transition or “renewal,” LVAD patients—particularly those receiving destination therapy—remain in a state of ongoing device dependence. This persistent awareness of technological reliance, combined with continuous exposure to device-related stressors such as alarms and driveline care, may contribute to sustained psychological burden. These differences highlight the importance of distinguishing LVAD populations from other cardiac cohorts when interpreting mental health outcomes and underscore the need for therapy-specific conceptual models.

The synthesis of available evidence suggests that depression and suicidality in LVAD recipients cannot be fully understood through traditional disease-centered models alone. Instead, they emerge from the interaction of patient-related, disease-related, device-related, and psychosocial factors, which evolve over time and may interact in non-linear ways. This perspective supports a shift toward longitudinal and integrative approaches to understanding psychological outcomes in this population.

These findings are further supported by recent integrative models of psychosocial adaptation in cardiovascular disease. Jackson et al. proposed a comprehensive framework emphasizing the interaction between emotional responses, cognitive appraisal, social context, and behavioral adaptation following major cardiovascular events [99]. Their work highlights that psychological outcomes are not solely determined by disease severity but are shaped by patients’ perceptions, coping strategies, and available social support. This perspective aligns closely with the challenges faced by LVAD recipients, in whom long-term device dependence, altered identity, and caregiver dynamics play a central role. Importantly, this model also indicates the need for multidisciplinary and longitudinal approaches to care, integrating psychological, social, and medical domains to improve overall outcomes [99,100].

Despite increasing recognition of these challenges, the current evidence base remains limited. Much of the available literature is derived from single-center or registry-based studies, with relatively short follow-up and limited use of standardized psychiatric assessment tools. This restricts the ability to characterize the temporal evolution of depression and suicidality and to identify critical periods of vulnerability. Prospective longitudinal studies spanning the full continuum of LVAD care—from candidacy assessment through long-term support—are therefore essential to advance the field [14,101].

The development of dedicated suicide risk registries in LVAD populations represents an additional avenue for advancing knowledge. Such registries could facilitate more accurate estimation of incidence, identification of high-risk subgroups, and evaluation of preventive strategies. Evidence from broader suicide prevention research indicates that systematic monitoring and early identification of at-risk individuals are associated with improved outcomes, suggesting that similar approaches may be applicable in this setting [102,103].

There is also a need for rigorously designed clinical trials evaluating both pharmacological and psychotherapeutic interventions in LVAD recipients. The unique clinical context—including the interaction between antidepressant therapy and anticoagulation, as well as the impact of device-related complications—necessitates tailored study designs that account for these factors [23,73]. In parallel, emerging psychotherapeutic approaches focusing on psychological flexibility, adaptation to chronic illness, and coping with long-term device dependence warrant further investigation.

Digital mental health technologies represent a promising area of development, offering opportunities for continuous monitoring and early detection of psychological distress. Approaches such as ecological momentary assessment, wearable monitoring, and app-based interventions may be particularly well suited to LVAD populations, in whom mobility limitations and healthcare access barriers are common. Growing evidence supports the potential of these tools to improve engagement, adherence, and clinical outcomes in chronic disease populations [104,105].

Finally, the role of caregivers remains a critical yet underexplored dimension. The bidirectional relationship between patient and caregiver distress suggests that psychological outcomes should be considered within a dyadic framework rather than in isolation. Future research should therefore prioritize caregiver-focused interventions and the development of integrated models addressing both patient and caregiver needs. In addition, the creation of LVAD-specific psychosocial assessment tools—rather than reliance on instruments developed for other populations—may improve risk stratification and clinical decision-making in this unique group [64,106].

In summary, depression and suicidality in LVAD patients represent complex, multifactorial phenomena that extend beyond traditional clinical boundaries. Their effective understanding requires integration of biological, psychological, and social perspectives, as well as recognition of device-specific factors. Advancing the field will depend on the development of longitudinal research frameworks, tailored assessment tools, and interventions that reflect the unique challenges of life with mechanical circulatory support. Key studies investigating these issues are summarized in Table 4.

## 13. Limitations

This review and the underlying body of literature on depression and suicidality in patients undergoing advanced cardiac therapies are subject to several important limitations, which should be carefully considered when interpreting the findings.

### 13.1. Limitations of the Available Evidence Base

A major limitation of the current literature is the predominance of single-center and registry-based studies, often involving relatively small sample sizes. This restricts statistical power and limits the generalizability of findings across broader populations [23]. Although the ASSIST-ICD study represents the largest prospective dataset on suicidality in LVAD recipients, including 494 eligible patients, it was conducted within a single country and healthcare system, which may limit its external validity [29].

In addition, there is substantial heterogeneity across studies with respect to patient populations, device types, study designs, duration of follow-up, and the instruments used to assess depression and suicidality. This variability complicates direct comparison between studies and limits the ability to draw consistent conclusions.

The measurement of depression and suicidality also varies considerably, with differences in screening tools, diagnostic criteria, and thresholds. Furthermore, somatic symptoms associated with advanced heart failure—such as fatigue, sleep disturbance, and reduced functional capacity—may overlap with depressive symptomatology, potentially leading to misclassification or overestimation of depression prevalence.

Another important limitation is the likely underreporting of suicidality. Stigma surrounding mental health, inconsistent screening practices, and variability in clinical documentation may contribute to under-recognition of suicidal ideation and behavior. This issue is particularly relevant in LVAD populations, where suicide may occur through mechanisms such as device disconnection that are not always clearly classified or reported as intentional self-harm.

Finally, there is a lack of prospective longitudinal studies incorporating systematic and repeated psychiatric assessments from the pre-implantation phase through long-term follow-up. Most available data are derived from cross-sectional studies or studies with relatively short follow-up periods, limiting the ability to capture the dynamic trajectory of psychological outcomes over time.

### 13.2. Population and Generalizability Limitations

The majority of studies in this field have been conducted in predominantly male, middle-aged, Western populations. This raises important concerns regarding the generalizability of findings to women, older adults, and racially and ethnically diverse populations, who may exhibit different patterns of psychological vulnerability, coping strategies, and access to mental health care.

In addition, differences in cultural perceptions of illness, mental health, and end-of-life decision-making may further influence both the expression and reporting of psychological distress, introducing additional variability across healthcare settings.

### 13.3. Methodological Limitations of the Present Review

This narrative review itself has inherent methodological limitations. Although a structured search strategy was employed, the study design is non-systematic and therefore subject to selection bias. The absence of a formal meta-analysis reflects the heterogeneity of available studies and limits the ability to perform quantitative synthesis or establish pooled estimates.

Furthermore, while this review proposes conceptual frameworks and clinical models—including multidimensional risk factors and integrated management pathways—these are based on synthesis of existing evidence rather than prospective validation. As such, their clinical applicability requires confirmation through future high-quality studies.

### 13.4. Implications for Future Research

Taken together, these limitations highlight the need for large-scale, prospective longitudinal studies with standardized assessment of depression and suicidality across the continuum of advanced cardiac care. There is also a need for interventional trials evaluating targeted pharmacological, psychotherapeutic, and multidisciplinary strategies in LVAD populations.

Future research should prioritize diverse and representative patient populations, standardized outcome measures, and the development of LVAD-specific psychosocial assessment tools to enhance both clinical applicability and generalizability.

## 14. Conclusions

Depression and suicidality are significant yet underrecognized components of advanced heart failure, particularly in patients supported with LVADs. These conditions arise from a complex interaction of biological, psychological, device-related, and social factors, with LVAD recipients facing unique risks related to device dependence and access to self-harm means. Current evidence remains limited and heterogeneous, and the integration of mental health into routine cardiovascular care is still inconsistent. Future progress will depend on improved longitudinal data, standardized assessment of suicide risk, and the development of targeted, LVAD-specific interventions to better address the needs of this vulnerable population.

## 15. Take-Home Messages

- Depression is common in advanced heart failure and LVAD patients and significantly affects outcomes.
- Suicidal ideation is not rare and is likely underrecognized in this population.
- LVAD therapy introduces unique psychological and ethical challenges.
- Device dependence and access to self-harm means increased suicide risk.
- Depression and suicidality are multifactorial (biological, psychological, and social).
- Psychological distress changes over time and requires ongoing monitoring.
- Routine screening for depression and suicidality should be standard practice.
- Pre-existing psychiatric conditions require management, not exclusion.
- Multidisciplinary care is essential for optimal outcomes.
- Treatments must be individualized and adapted to LVAD-specific risks.
- Early palliative care and caregiver support improve patient care.
- Further research is needed to improve risk assessment and management.

## Figures and Tables

**Table 1:** Prevalence of depression and suicidality across advanced cardiac therapy populations.

Population	Depression Prevalence	Suicidal Ideation	Suicide Attempts/Completion	Key References
General heart failure population	~20–25%	Not consistently reported	Not consistently reported	Zeng et al. [7]
Advanced heart failure (NYHA III–IV)	Up to 42%	Increased risk (not consistently quantified)	Not consistently reported	Polikandrioti & Tsami [8]
LVAD candidates (pre-implant)	~40–41%	Not consistently reported	Not consistently reported	Yost et al. [24]
LVAD recipients (post-implant)	~15–20%	~12%	~2% over ~18 months	Alnsasra et al. [23]; ASSIST-ICD [29]
Heart transplant recipients	~21.6%	Not consistently reported	Rare, not well quantified	Loh et al. [36]
ECMO survivors	~24%	Not consistently reported	Not consistently reported	Demory et al. [41]
Chronic illness populations (general)	Variable	Increased risk compared to general population	Higher than general population	Nafilyan et al. [16]

**Table 2:** Validated screening tools for depression and suicidality in patients with advanced cardiac disease and LVAD support.

Tool	Domain Assessed	Format/Items	Cut-Off/Interpretation	Advantages	Limitations in LVAD/HF Population
Patient Health Questionnaire-9 (PHQ-9)	Depression	9-item self-report	≥10 suggests moderate depression	Brief, widely validated, guideline-supported	Somatic symptoms (fatigue, sleep) may overlap with HF
Hospital Anxiety and Depression Scale (HADS)	Anxiety & Depression	14 items (7 + 7)	≥8 per subscale suggests caseness	Minimizes somatic symptom bias, suitable for cardiac patients	Less sensitive for severe depression
Beck Depression Inventory-II (BDI-II)	Depression severity	21-item self-report	≥14 mild, ≥20 moderate depression	Comprehensive, widely used in LVAD studies	Longer, includes somatic items
Columbia Suicide Severity Rating Scale (C-SSRS)	Suicidal ideation & behavior	Structured interview	No fixed cut-off; graded severity	Gold standard, detailed risk stratification	Requires training, more time-consuming
Ask Suicide-Screening Questions (ASQ)	Suicide risk	4-item rapid screening	Any “yes” requires further assessment	Quick, feasible in clinical settings	Limited depth; requires follow-up assessment
Stanford Integrated Psychosocial Assessment for Transplantation (SIPAT)	Psychosocial risk (pre-implant)	Multidomain clinician-rated tool	Higher scores = higher risk	Specifically relevant for LVAD/transplant evaluation	Requires trained personnel, not a screening tool per se

**Table 3:** Management strategies for depression and suicidality in patients with LVAD support.

Intervention Category	Examples	Evidence/Clinical Benefit	Special Considerations in LVAD Patients
Pharmacological treatment	Selective serotonin reuptake inhibitors (SSRIs)	Effective for depression; generally safe in cardiac populations	Increased bleeding risk due to concomitant anticoagulation; careful monitoring required
Psychotherapy	Cognitive behavioral therapy (CBT), Acceptance and Commitment Therapy (ACT), supportive therapy	Improves depression, coping, and quality of life	Requires adaptation to chronic device dependence and physical limitations
Multidisciplinary care	Cardiology–psychiatry collaboration, LVAD coordinators, social workers	Improves detection of psychological distress and overall outcomes	Coordination across specialties is essential; resource-dependent
Palliative care integration	Early involvement of palliative care teams	Reduces symptom burden, improves quality of life, supports advance care planning	Often introduced late; should be integrated early in LVAD pathway
Caregiver support interventions	Education, counseling, caregiver support programs	Reduces caregiver burden and indirectly improves patient outcomes	Dyadic relationship between patient and caregiver is critical
Digital mental health interventions	Telepsychiatry, app-based monitoring, remote counseling	Improves access to care; may enhance adherence and early detection	Evidence still emerging; requires patient engagement and digital literacy
Suicide risk management	Structured screening (e.g., C-SSRS, ASQ), safety planning, psychiatric referral	Enables early identification and prevention of suicidal behavior	Must include discussion of device-related means (e.g., disconnection) with careful and ethical communication

**Table 4:** Key studies on depression and suicidality in LVAD and advanced cardiac therapy populations.

Study (First Author, Year)	Population/Sample Size	Study Design	Key Findings	Clinical Relevance
Charton M et al., 2020 (ASSIST-ICD) [29]	LVAD recipients (*n* = 494)	Multicenter cohort	~2% suicide attempts/completions over ~18 months; unique methods (device disconnection, overdose)	Demonstrates elevated suicide risk and device-specific mechanisms
Yost G et al., 2017 [24]	LVAD candidates and recipients (*n* ≈ 120)	Observational	~41% moderate depression pre-implant; reduction post-implant	Highlights importance of pre-implant screening and dynamic symptom change
Alnsasra H et al., 2023 [23]	LVAD recipients	Narrative review/cohort data synthesis	~15–20% depression post-implant; ~12% suicidal ideation	Confirms persistent psychological burden despite physical improvement
D’Aoust RF et al., 2021 [25]	LVAD recipients	Observational	Strong association between sleep disturbance and depression	Identifies modifiable risk factors for intervention
DeFilippis EM et al., 2020 [18]	LVAD recipients (*n* = 15,403)	Registry analysis	~20.5% had psychosocial risk factors; associated with worse outcomes	Supports need for structured psychosocial assessment
Loh AZH et al., 2020 [36]	Heart transplant recipients (*n* = 2169)	Meta-analysis	Depression prevalence ~21.6%; PTSD and anxiety also common	Demonstrates psychological burden across advanced therapies
Fernando SM et al., 2022 [40]	ECMO survivors	Population-based cohort	Increased incidence of new psychiatric disorders (HR 1.24)	Highlights long-term mental health impact of critical illness
Nafilyan V et al., 2022 [16]	General population (*n* ≈ 47 million)	Retrospective cohort	Chronic illness strongly associated with increased suicide risk	Provides broader context for suicidality in medical populations

LVAD: left ventricular assist device; ECMO: extracorporeal membrane oxygenation. Studies vary in design, population characteristics, and outcome definitions, limiting direct comparability. Reported rates of depression and suicidality may be influenced by differences in assessment tools and potential underreporting.

## Data Availability

No new data were created or analyzed in this study.
